# Oncological outcome after local treatment for early stage rectal cancer

**DOI:** 10.1007/s00464-021-08308-1

**Published:** 2021-02-05

**Authors:** Caroline D. M. Witjes, Abhilashaben S. Patel, Aniruddh Shenoy, Stephen Boyce, James E. East, Christopher Cunningham

**Affiliations:** 1 Department of Colorectal Surgery, Churchill Hospital, Oxford University Hospital, NHS Foundation Trust, Old Road, Headington, OX3 7LE Oxford UK; 2 Translational Gastroenterology Unit, Nuffield Department of Medicine, John Radcliffe Hospital, University of Oxford, Oxford, UK; 3 Oxford NIHR Biomedical Research Centre, University of Oxford, Oxford, UK

**Keywords:** Early rectal cancer, Local treatment, Polypectomy (EMR/ESD), TEM, TAMIS

## Abstract

**Background:**

Treatment of early rectal cancer is evolving towards organ-preserving therapy which includes endoscopic resection and transanal approaches. We aimed to explore the role of local treatments such as endoscopic polypectomy (Endoscopic Mucosal Resection (EMR) or Endoscopic submucosal dissection (ESD)) and transanal endoscopic microsurgery/ transanal minimal invasive surgery (TEM/TAMIS) in patients who had early rectal cancer. We considered these outcomes alongside conventional major surgery using total mesorectal excision (TME) for early stage disease.

**Methods:**

All patients identified at MDT with early stage rectal cancer at our institution between 2010 and 2019 were included. Long-term outcomes in terms of local recurrence, survival and procedure-specific morbidity were analysed.

**Results:**

In total, 536 patients with rectal cancer were identified, of which 112 were included based on their pre-operative identification at the MDT on the basis that they had node-negative early rectal cancer. Among these, 30 patients (27%) had the lesion excised by flexible endoscopic polypectomy techniques (EMR/ESD), 67 (60%) underwent TEM/TAMIS and 15 (13%) had major surgery. There were no differences in patient demographics between the three groups except for TEM/TAMIS patients being more likely to be referred from another hospital (p < 0.001) and they were less active (WHO performance status p = 0.04). There were no significant differences in overall survival rates and cancer-specific survival between the three treatment groups. The 5-year overall survival rate for endoscopic polypectomy, TEM/TAMIS or major resection was 96% versus 90% and 88%, respectively (p = 0.89). The 5- year cancer-specific survival rate was 96%, versus 96% and 100%, respectively (p = 0.74).

**Conclusion:**

Endoscopic polypectomy by EMR/ESD is an appropriate local treatment for early stage rectal cancer in selected patients. It is possible to achieve good oncological outcomes with a polypectomy similar to TEM/TAMIS and major surgery; however, a multidisciplinary approach is necessary enabling close surveillance and the use of adjuvant radiotherapy.

Rectal cancer has conventionally been treated with major surgical resection based on the principles of total mesorectal excision (TME) [1]. Although this approach has considerably reduced local recurrence and improved survival, it is associated with significant risk of morbidity including sexual, urinary or bowel dysfunction [1, 2]. Over the last few years, there has been increasing interest in developing organ-preserving approaches which would not subject patients to these risks [1, 3–5].

Initial reports of using an organ-preserving approach were focussed on patients treated with neo-adjuvant chemoradiation. Those patients with a good clinical response to this treatment were offered a watch and wait policy or the rectal cancer was treated with local resection which often utilised trans-anal endoscopic microsurgery (TEM)/ trans-anal minimal invasive surgery (TAMIS), being effective at removing small cancers [4, 6]. Similarly, with the development of endoscopic mucosal resection (EMR) and endoscopic submucosal dissection (ESD), it is now possible to resect more advanced polyps and early cancers safely [7–10]. Both techniques are increasingly used for early rectal cancer and a number of studies have demonstrated that it is also safe to perform a TEM/ TAMIS after a polyp resection [11–15].

The aim of this study is to describe outcomes in patients with early stage rectal cancer who underwent treatment with either endoscopic polypectomy via EMR or ESD, trans-anal approached surgery (TEM/TAMIS) or a major surgical resection. Given the availability of expertise in all three of these techniques at our institution, this study seeks to demonstrate how the different treatment modalities are related to see whether it can guide clinical decision-making to achieve the optimal outcome.

## Material and methods

### Clinical characteristics

Oxford University NHS Foundation Trust is a large tertiary referral centre for rectal cancer in the United Kingdom. The details of all patients discussed in the multidisciplinary meeting are recorded in a prospective electronic database which was utilised to identify all patients with early stage rectal cancer (T1/T2 pre-operative), no suspected or enlarged lymph nodes (N0), and no metastatic disease (M0), treated in the hospital between February 2010 and December 2019. Patients with T2 rectal cancer pre-operative (as recorded at MDT) and T2 or higher post-operative were excluded for this study.

Clinical data regarding patient characteristics, diagnostic tests, tumour characteristics, histology reports, TNM stage (7th or 8th edition regarding year of diagnosis), treatment, and follow-up were extracted from this database and were analysed retrospectively [16, 17].

We included patients in this study if they were diagnosed with an adenocarcinoma and were suitable for either local treatment and/or major surgery based on their pre-operative or post-polypectomy histology report. All patients had pre-treatment cancer work-up with a digital examination, an endoscopy with high definition instrument, narrow band imaging or dye-spray as indicated, an MRI pelvis and/or endorectal ultrasonography and an CT thorax, abdomen and pelvis.

### Treatment

Treatment decision was made by the patient and clinician based on MDT guidance following the valid guideline at time of diagnosis. The patient and the patient’s wishes were considered when determining which patient should undergo which treatment; largely the decision of the MDT was followed. The reason behind treatment decisions was not always documented in detail and therefore could not be analysed in this study.

Organ-preserving local endoscopic treatment included all endoscopic polypectomy techniques including EMR and ESD. Organ-preserving surgical treatment includes TEM and TAMIS. Major TME surgery included low anterior resection (LAR) or abdominal perineal resection (APR). Treatment-related complications were reported up to 30-days post procedure.

If patients underwent more than one treatment e.g., polypectomy followed by TEM/TAMIS and/or TME surgery, they were analysed according to their final treatment.

The resection was recorded as R0 if there was a definitive microscopic resection margin of 1 mm or more and R1 if the microscopic resection margin was less than 1 mm or if it was not possible to comment on the distance to peripheral margins because of the specimen being too fragmented. The first endoscopy site check needed to show a healthy scar without signs of residual tumour if patients we considered for no further treatment but follow-up only.

### Follow-up

Patients had close follow-up for at least 5 years with a clinical examination, flexible sigmoidoscopy and MRI every 4 months for 2 years, after that they had a sigmoidoscopy and an MRI twice annually for at least 3 years. This was combined with annual CT thorax, abdomen and pelvis for 3 years and colonoscopy as indicated by national guidance. Recurrence was defined as local recurrence or appearance of metastatic disease.

### Statistical analysis

Variables were compared using the Mann–Whitney U test, Kruskal–Wallis H or with the χ^2^-test, as appropriate. Statistical significance was considered at a p-value < 0.05. Survival curves were drawn using the Kaplan–Meier method. Differences between Kaplan–Meier curves were tested using the log-rank test. All analyses were performed using the Statistical Package for Social Sciences 26.0 (SPSS, Chicago, IL, USA).

### Consent

This study compliances with ethical standards. Requirements for informed consent were waived because analysis were done on existing data without any potential influence on participants. The study is registered, and it is conducted as an approved audit by the Oxford University Hospitals, NHS Foundation Trust.

## Results

### Study population

**Fig. 1:**
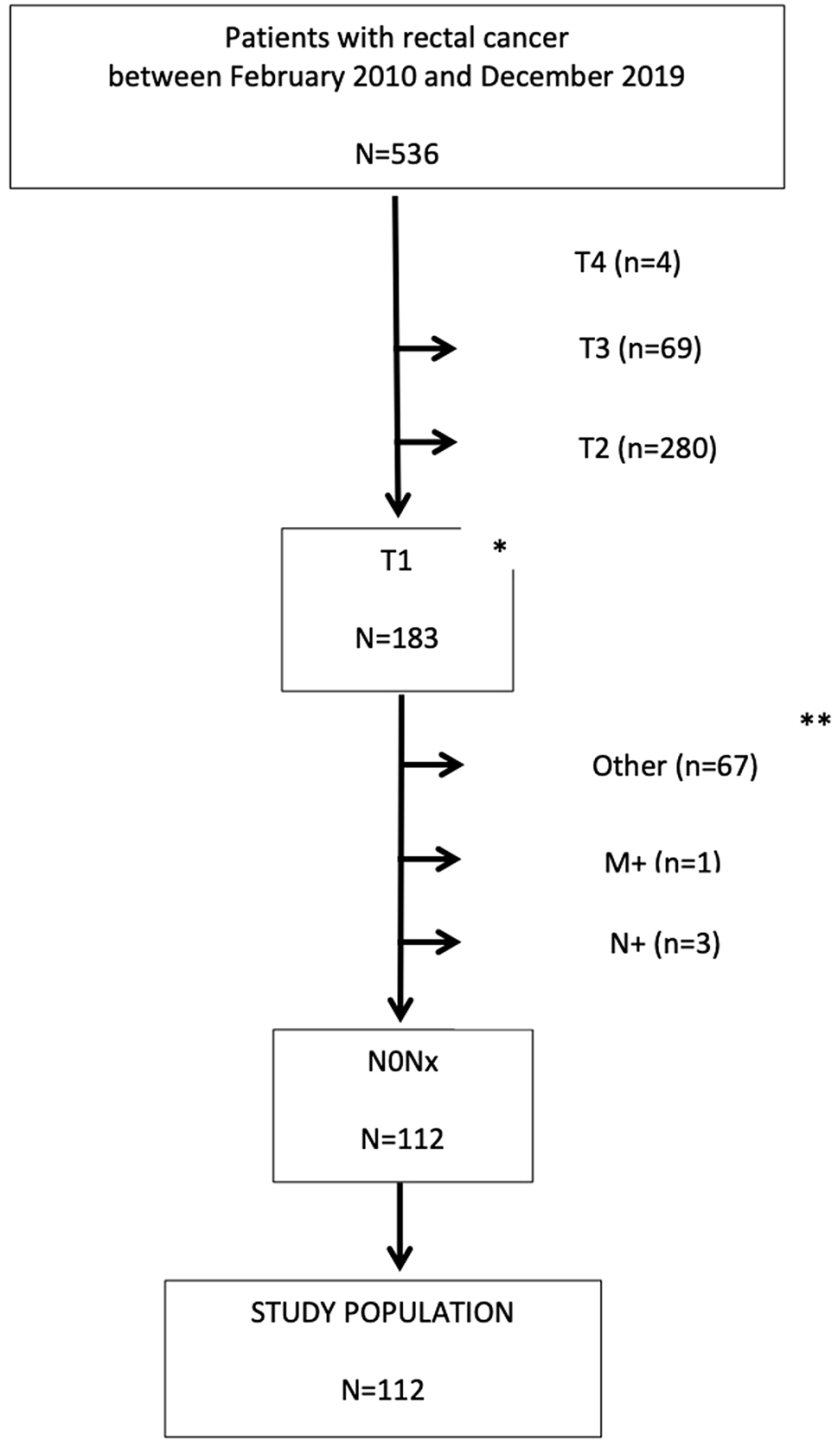
Study population flow diagram. *Including T1/T2 pre-operatively, definitive T2 pre- and post-operative were excluded. **e.g. neuro-endocrine tumour, squamous cell cancer, cancer presented in ulcerative colitis, presented with a second bowel tumour at the same time, presented with a colovesical fistula not cancer related, no treatment in our hospital only MDT opinion, died after diagnosis, before treatment could be discussed of another cause, presented with cancer while having familial adenomatous polyposis (FAP) or a not representative biopsy showing high grade dysplasia which was not confirmed as a malignancy after resection

In total, 536 patients with rectal cancer were identified, of which 112 were included in this study as shown in Fig. 1. The baseline characteristics of the study population are summarised in Table 1.

**Table 1:** Summary of patient characteristics of patients with early rectal cancer

Patient characteristics	Study population (*n* = 112)*
Age (years)	68 (35–92)
World Health Organization (WHO) Classification	
Fully active or no heavy physical work	101 (90)
1/2 a day or more in bed	11 (10)
American Society of Anaesthesiologists (ASA) Classification	
Normal health or mild systemic disease	106 (95)
Severe systemic disease	6 (5)
Male	68 (61)
Referral from another hospital	56 (50)
Distance to anal verge (cm)	7 (1–18)
Endoscopic lesion size (mm)	20 (6–110)
Treatment	
Local endoscopic—polypectomy (EMR/ESD)	30 (27)
Local surgical—TEM/TAMIS	67 (60)
Major surgical—LAR or APR	15 (13)

*Number(%) or median(range)

### Treatment

Over the study period, all three treatment modalities were offered with no trends suggesting a change in practice in favour of a particular treatment. Details of patients in the three treatment groups are presented in Table 2. Of the 21 patients who had an endoscopic en bloc resection six (29%) had an EMR, six (29%) had an ESD and nine (42%) had an en bloc resection with the diathermia snare technique after pre-lifting-injected. All patients shown in the second column, undergoing endoscopic polypectomy, had an endoscopic radical resection where all macroscopically visible tumour or polyp was removed. An R1 polypectomy refers to the presence of tumour less than 1 mm from the margin after resection or if the specimen is too fragmented to identify a true resection margin, this was the case in 53% (16/30). Of those patients that had a R1 polypectomy, one patient opted for adjuvant radiotherapy despite no evidence of lymphovascular invasion; the remainder preferred a watch- and wait approach. None of the patients had a readmission after polypectomy.

**Table 2:** Summary of characteristics of patients related to treatment for early rectal cancer

Patient characteristics	Endoscopic Polypectomy patients (*n* = 30)*	TEM/TAMIS patients (*n* = 67)*	LAR or APR patients (*n* = 15)*	*p* value
Age (years)	72 (45–88)	67 (42–92)	62 (35–67)	0.10
World Health Organization (WHO) Classification				**0.04**
Fully active or no heavy physical work	24 (80)	62 (93)	15 (100)	
1/2 a day or more in bed	6 (20)	5 (7)	0 (0)	
American Society of Anaesthesiologists (ASA) Classification				0.41
Normal health or mild systemic disease	28 (93)	63 (94)	15 (100)	
Severe systemic disease	2 (7)	4 (6)	0 (0)	
Male	18 (60)	43 (64)	7 (47)	0.46
Referral from another hospital	8 (27)	45 (67)	3 (20)	** < 0.01**
Distance to anal verge (cm)	9 (1–18)	6 (1–16)	8 (2–15)	0.08
Endoscopic lesion size (mm)	20 (6–110)	25 (6–100)	17 (7–80)	0.88
Previous treatment				N.A.^
Polypectomy and TEM/TAMIS	N.A	N.A	3 (20)	
Straight to major resection	N.A	N.A	12 (80)	
First endoscopic intervention				** < 0.001**
Biopsy	0 (0)	60 (90)	9 (60)	
En bloc resection (EMR, ESD or other)	21 (70)	6 (9)	5 (33)	
Polypectomy	5 (17)	1 (2)	0 (0)	
Piecemeal	4 (13)	0 (0)	1 (7)	
Histology differentiation grade				0.70
Well	3 (10)	13 (19)	2 (13)	
Moderate	25 (83)	52 (78)	12 (80)	
Poor	2 (7)	2 (3)	1 (7)	
Lymphovascular invasion	2 (7)	12 (18)	4 (27)	0.16
Radical resection				N.A.^
R0	14 (47)	57 (85)	15 (100)	
R1	16 (53)	10 (15)	0 (0)	
Margin < 1 mm	11 (69)	7 (70)	0 (0)	
Margin could not be defined or margin involved	5 (31)	3 (30)	0 (0)	
Tumour free margin (mm)	2 (1–16)	6 (1–19)	N.A	N.A.^
No tumour residue in resection on histology	N.A	N.A	6 (40)	N.A.^
Treatment-related complications	0 (0)	5 (7)	2 (13)	** < 0.001**
Adjuvant therapy	1 (3)	8 (12)	2 (13)	0.14

*Number (%) or median (range)

^Variable could not be tested statistically

In third column in Table 2, the data from the patients who had a TEM/TAMIS are summarised. Six of these patients had undergone an endoscopic en bloc resection, three (50%) had an EMR, the other three (50%) had another type of en bloc resection, none specified. Amongst the 10 patients (15%) that had a R1 resection after TEM/TAMIS surgery, six patients had chemoradiotherapy, two patients had radiotherapy alone and two preferred a watch- and wait policy. Overall, five patients (7%) had a complication which included: pneumonia treated with antibiotics, presacral abscess treated with antibiotics, acute kidney injury treated conservatively and prolonged post-operative antibiotics because of dehiscence of the rectal wound after a full TEM thickness resection. In one patient, the wound could not be closed via TEM and conversion to laparoscopy for sewing the full thickness defect was needed.

The characteristics of the patients who underwent a major resection are summarised in fourth column in Table 2. There were two patients who had major surgery and were subsequently treated with adjuvant chemotherapy as they had nodal disease; however, there was no residual tumour in the rectum. In one of these two patients, suspicion of nodal disease was raised because of FDG uptake in a small sigmoid mesentery node on CT-PET scan 4 months after polypectomy during staging. The FDG uptake in this node was within the ranges of the uptake of a reactive node. However, concerns for malignancy were shared and therefore the patient proceeded to TME resection. Two patients (13%) had a post-operative complication. One patient had lung atelectasis treated conservatively and one patient had an anastomotic leak and needed a re-operation. This patient already had an ileostomy and was managed with a wash-out and re-suturing of the anastomosis. Four patients (27%) had a primary ileostomy. All patients had a reversal of their ileostomy, in one patient this was complicated and a second operation with adhesiolysis was needed two days after the ileostomy reversal.

As shown in column five in Table 2, the three treatment groups had similar patient characteristics in terms of gender (p = 0.46), ASA (p = 0.41), age (p = 0.10), distance from anal verge (p = 0.08), tumour size (p = 0.88), histology grade (p = 0.70) and microscopic lymphovascular invasion (p = 0.16). The patients who underwent a TEM/TAMIS were more likely to be referred from another hospital (p < 0.001) and were less active (WHO performance status p = 0.04) compared to the patients who underwent a polypectomy or a major resection. Treatment-specific data are not similar and are, therefore, statistically incomparable.

### Survival and recurrence

**Fig. 2:**
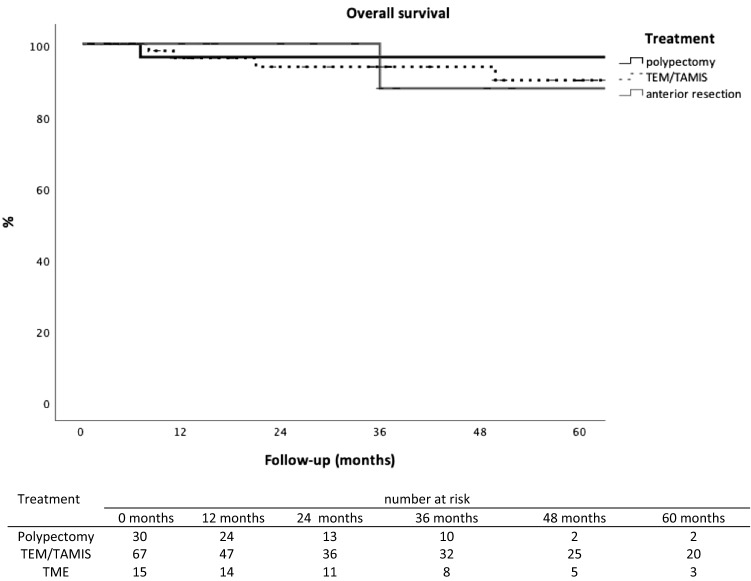
Overall survival rate related to treatment for early rectal cancer

There were no differences in 5-year overall survival of patients who had polypectomy, TEM/TAMIS or major resection (96% versus 90% versus 88%, respectively, p = 0.89) (see Fig. 2).

After polypectomy, two patients developed recurrent disease despite having had a R0 polypectomy and no lymphovascular invasion. One of these patients had local recurrence seven months after polypectomy which was treated with radiotherapy as further resection with a TEM/TAMIS procedure was not possible due to the location of the tumour. Although there was no recurrence or regrowth, this patient died 14 months after radiotherapy of a non-cancer-related cause. The other patient developed metastatic deposits in the liver 14 months after polypectomy.

Of the patients who underwent a TEM/TAMIS, three patients had recurrence of disease. Two patients had local recurrence after 11 and 23 months, respectively. The patient with recurrence after 11 months had prior a R0 resection with microscopic lymphovascular invasion and the recurrence was treated with chemoradiotherapy only. The patient with recurrence after 23 months (R0 resection and no lymphovascular invasion) had a PET suspected tumour deposit in the sacral area and therefore had an APR after neo-adjuvant chemoradiotherapy, however, developed lung metastases four years after the APR (T3N0). During surveillance, the third patient presented 11 months after treatment (R1 resection with lymphovascular invasion) with liver and lung metastasis without a local recurrence.

**Fig. 3:**
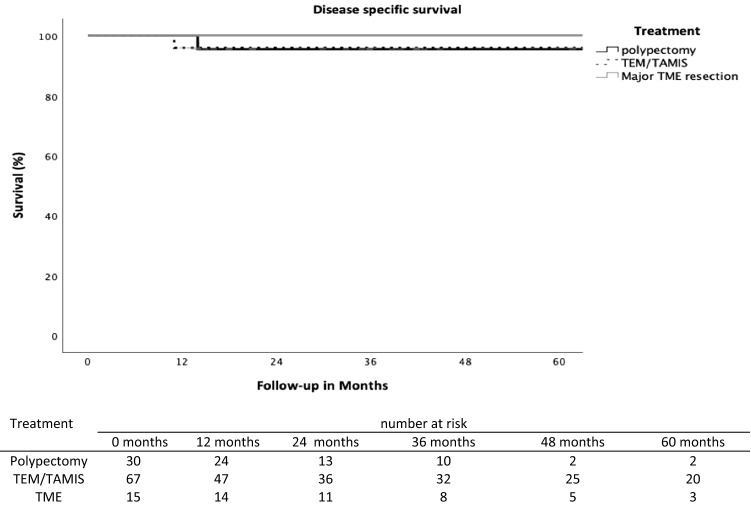
Cancer specific survival rate related to treatment for early rectal cancer

The cancer-specific 5-year survival rate for polypectomy was 96%, for TEM/TAMIS 96% and for major surgery 100%, respectively (p = 0.74) (Fig. 3).

## Discussion

Management of early stage rectal cancer continues to be a challenge for many multidisciplinary cancer teams. Decision-making post polypectomy, assessing the risk of luminal and mesorectal recurrence, not to mention distal disease, provokes considerable discussion. In this study, we primarily aimed to capture the outcomes after following the management of early stage rectal cancer by a selection of approaches available to all MDTs. We considered endoscopic treatment of early cancer by EMR/ESD/standard polypectomy, along with to surgical treatment, which included either TEM/TAMIS or major surgery. The decision to adopt a particular treatment strategy is often subtle and based on several different factors including the location of the tumour, patient comorbidity and patient/surgeon preference. In our study, the three groups of patients showed similar patient and tumour characteristics. Our findings suggest that, in selected patients, long-term oncological outcomes after endoscopic excision of the polyp by EMR or ESD are no different to local excision and major resection surgery. Furthermore, it highlights that if endoscopic excision has been performed successfully, as defined by healthy scar at site check, further resection of the rectum by either TEM/TAMIS or major surgery may not be required unless there are adverse features that have been identified. This study reflects the reality of current clinical practice in a centre where all three options are available at expert level and describes the dilemmas encountered at MDT.

Several previous studies have examined oncological outcomes after local excision and radical surgery [3, 11–13, 15]. However, most of these studies have included both colonic and rectal cancer making it difficult to draw conclusions about rectal cancer specifically [13]. Others have investigated outcomes across all stages of cancer where local excision may not be sufficient to ensure complete removal of the tumour [3]. Reports of TEM/TAMIS surgery often include different histological types of cancer aside from adenocarcinoma or report other non-oncological outcomes making it difficult to draw conclusions about cancer-specific survival in these patients [18].

The results of our study suggest that oncological outcomes are similar across all three groups. A recent study has also examined local excision with TEM/TAMIS and compared outcomes to major surgery [19]. Patient characteristics, surgical outcome and survival rates were comparable to our study, as radical resection rates were 84% for TEM/TAMIS and 99% for major surgery; 5-year cancer-specific survival rates were 95% and 94%, respectively [19]. This demonstrates that, if oncological safety can be ensured, local excision with only a polypectomy could be considered sufficient treatment for early stage rectal cancer and may be attractive in patients who are not fit for surgical excision. Furthermore, our results highlight that a TEM/TAMIS resection of the polypectomy site or major surgical resection may be unnecessary if the scar is healthy with no evidence of regrowth and the MRI scan shows no distant lymphatic spread [14].

Despite the findings of this study showing promising outcomes for local therapy in rectal cancer, it is important to realise there are several limitations. Firstly, the number of patients in each treatment group is small and further investigation in larger cohorts of patients in a multicentre setting would be necessary to ensure the results can be generalised. Secondly, there will be selection bias as nearly half of the patients were referred from surrounding hospitals to our institution as it is a tertiary referral centre for TEM/TAMIS surgery and advanced endoscopic resection. Thus, bias for local therapy could have been introduced at an early stage in the decision-making process with patients opting for local excision instead of major surgery [1–3]. There is also a bias based on the age and comorbidity of the patient. A healthy patient, particularly if younger, is more likely to be offered major surgery, whereas an elderly patient with comorbidity may opt for local excision or a watch and wait policy to avoid the morbidity of major surgery. The vast majority of patients, however, fall in between these two extremes and are subjected to a detailed conversation in clinic where they are actively encouraged to participate in the final decision about treatment [4, 5, 20–22]. Hence, this study reflects the reality of current clinical practice where the decisions regarding surgery or local excision involve consideration of the patient’s wishes, the clinician’s expertise and the characteristics of the tumour. Thus, we did not seek to determine which factors contributed to decisions regarding which treatment arm the patient entered as it would be an impossible task when performed retrospectively. Prospective studies are already investigating these treatment modalities to determine the optimal treatment strategy [23].

Unfortunately, because of a heterogeneity in endoscopic and histology reports and as half of these patients were referred from another hospital, we were not able to analyse polyp features in the patients that were only treated with an endoscopic polypectomy. This is an important consideration for future studies and would allow comparison of outcomes based on polyp features if recording of endoscopy and histology reports could be standardised [24, 25]. Protocolised documentation would also help to inform the MDT discussion, especially when the discussion is about management after R1 polypectomy, where the treatment is usually tailored based on the available information [1].

Our study supports the notion that major surgery should be reserved for those with an incomplete excision, especially macroscopically incomplete and if adjuvant therapy cannot be given [14, 26–30]. However, local excision does not allow complete staging of the tumour as there is no reliable way to detect histologically positive lymph nodes [18, 31]. In our study, 7% of patients who had a polypectomy and 4% of TEM/TAMIS patients developed metastatic disease which is comparable to studies on major resection surgery with T1 disease where 10% patients develop distant metastases [32]. Close follow-up of all cohorts remains mandatory.

Amongst the patients found to have recurrent disease, three out of five patients (60%) developed metastatic disease without local recurrence. Interestingly, one of these patients who had a radical polypectomy developed a liver metastasis 14 months after the procedure. Our patient was unfit for liver surgery; however, there have been reports in the literature where a similar case presentation of a patient developing liver metastasis nine months after ESD was successfully treated with a liver resection and anterior resection [33]. However, no residual tumour was detected in the rectal cancer resection specimen nor in lymph nodes highlighting that these patients may have tumours that behaves differently, and local excision may not necessarily impair outcome. This most likely reflects inherent differences in tumour biology. However, as the overall recurrence rate in our study is low, we are not able to examine differences in outcome based on tumour biology.

In conclusion, this study demonstrates that it is possible to achieve good oncological outcomes in selected patients presenting with early stage rectal cancer with a polypectomy procedure, similar to patients with a TEM/TAMIS or major resection. If there is a wide involved margin after polypectomy, it may be possible to perform a wider local excision with TEM/TAMIS surgery, though often there is no residual tumour in the specimen, and therefore, this may not be always necessary. A local excision by TEM/TAMIS resection is, therefore, not warranted after a successful polypectomy. Further prospective studies need to determine which features of the tumour help to predict tumour biology and enable identification of patients who may require a more aggressive approach with major surgery.

## Abbreviations

TME: Total mesorectal excision
TEM: Trans-anal endoscopic microsurgery
TAMIS: Trans-anal minimal invasive surgery
EMR: Endoscopic muscosal resection
ESD: Endoscopic submucosal dissection
MDT: Multidisciplinary team
LAR: Low anterior resection
APR: Abdominal perineal resection
